# Supplementary material for: Polyphenol-Rich Extract from ‘Limoncella’ Apple Variety Ameliorates Dinitrobenzene Sulfonic Acid-Induced Colitis and Linked Liver Damage
Source: Int J Mol Sci. 2024 Mar 11;25(6):3210. doi: 10.3390/ijms25063210 (PMC10969867; doi:10.3390/ijms25063210)
Supplement: Supplementary file 1 [file ijms-25-03210-s001.zip › ijms-2846054-Figure SM.pptx]

## Slide 1
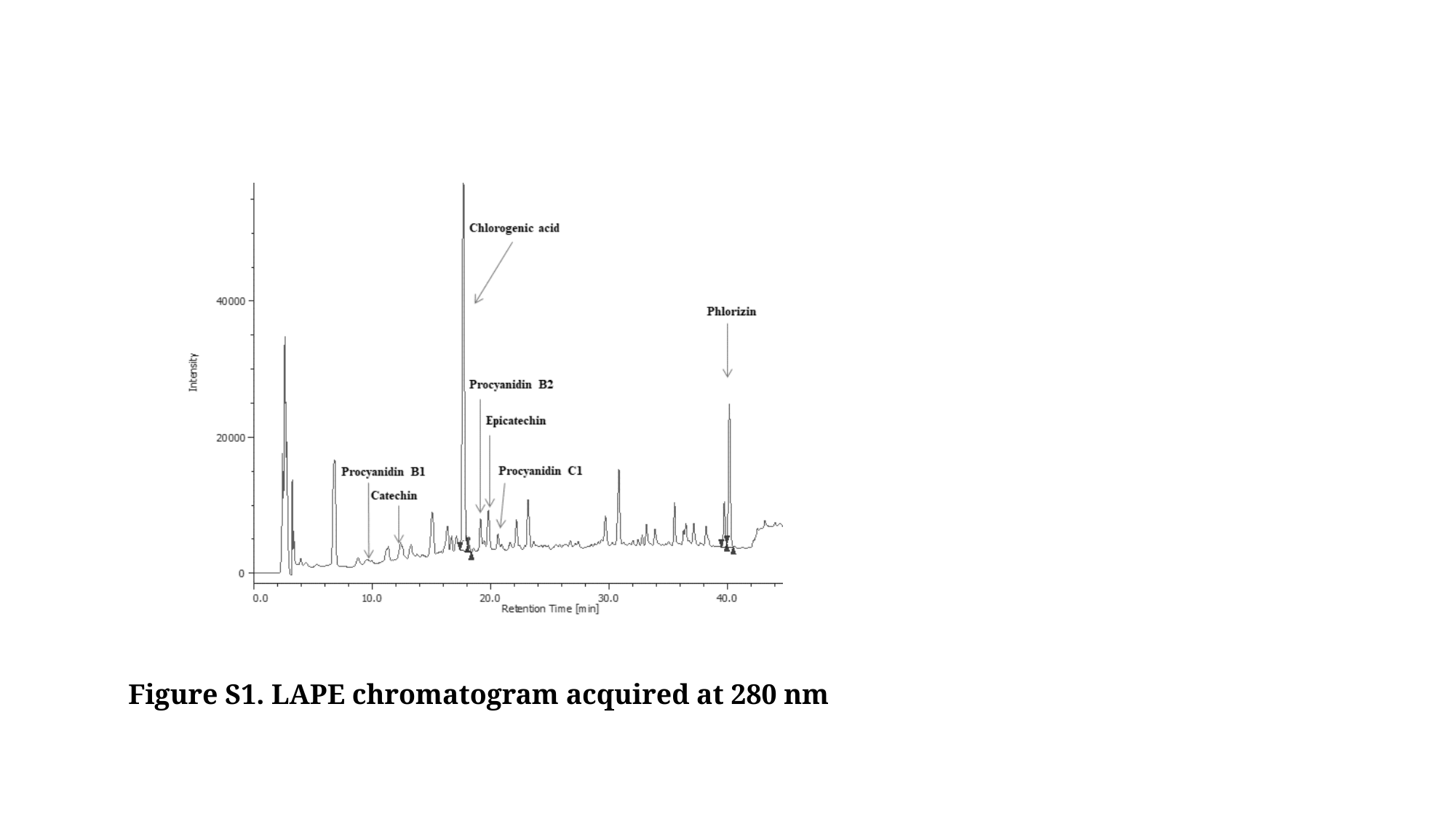

Figure S1. LAPE chromatogram acquired at 280 nm

## Slide 2
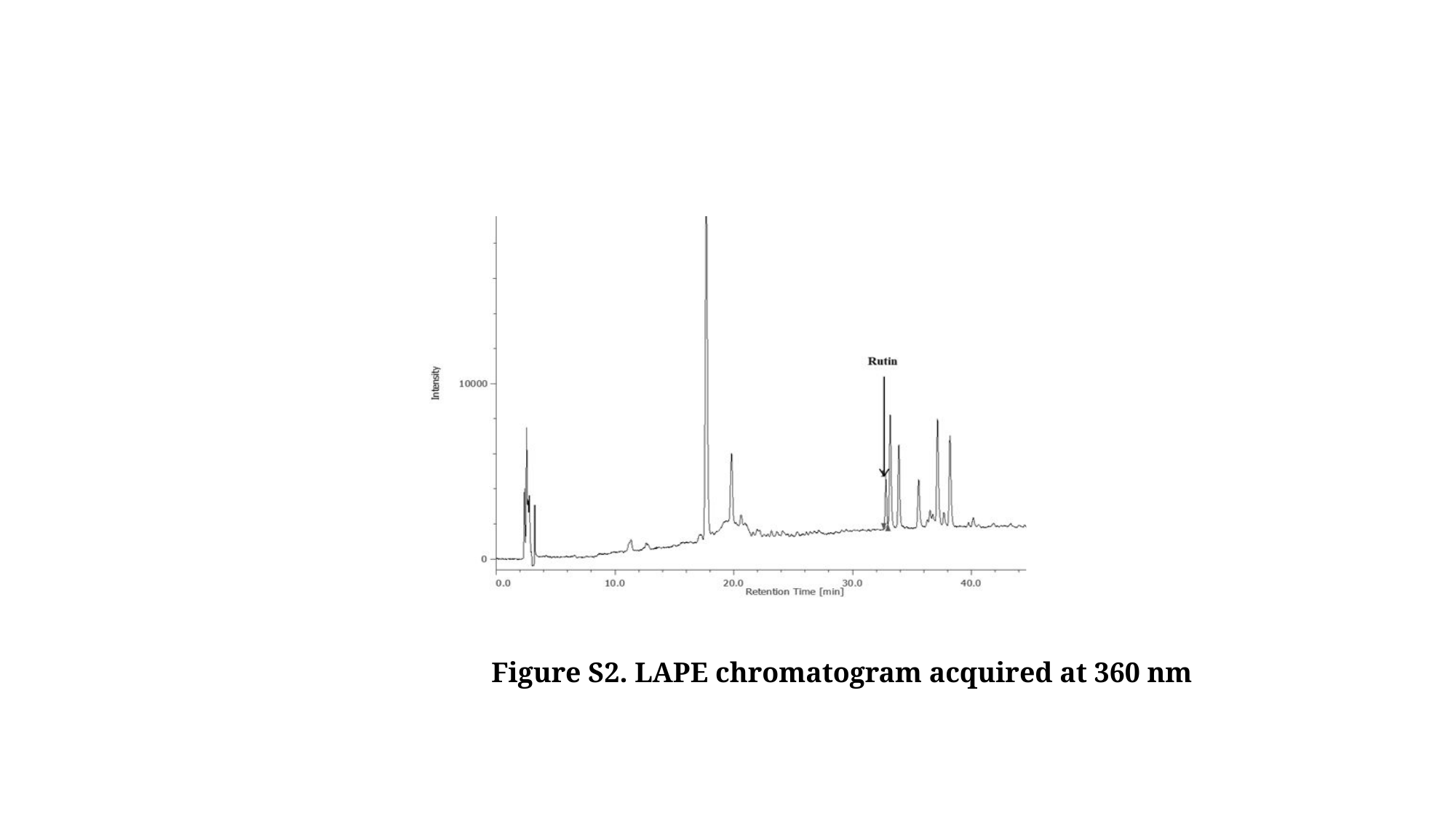

Figure S2. LAPE chromatogram acquired at 360 nm
